# Postoperative Outcomes and Complication Risk Factors in Zygomaticomaxillary Complex (ZMC) Fractures: A One-Year Retrospective Study of Surgical Management Approaches

**DOI:** 10.7759/cureus.98866

**Published:** 2025-12-10

**Authors:** Priyadharsana PS, Samuel Sugantharaj L, Prabhusankar K, Effie Edsor, Vigneshwar S, Vignesh K

**Affiliations:** 1 Department of Oral and Maxillofacial Surgery, RVS Dental College and Hospital, Coimbatore, IND; 2 Department of Oral and Maxillofacial Surgery, AB Shetty Memorial Institute of Dental Sciences, Nitte, Mangalore, IND

**Keywords:** facial trauma, open reduction and internal fixation, postoperative complications, retrospective study, zygomaticomaxillary complex

## Abstract

Background

Zygomaticomaxillary complex (ZMC) fractures are among the most frequently encountered midfacial injuries, often resulting from high-energy trauma such as road traffic accidents or assaults. Effective management requires a balance between aesthetic reconstruction and functional rehabilitation. Despite advancements in surgical techniques, postoperative complications remain a concern. This study aims to evaluate demographic patterns, etiological factors, fracture distribution, and surgical approaches, and to identify predictors of postoperative complications using multivariate analysis and survival methods.

Methods

A retrospective observational study was conducted on 77 patients surgically treated for ZMC fractures between 2021 and 2024. Data regarding patient demographics, mechanism of injury, fracture classification, incision type, and postoperative complications were extracted from hospital records. Chi-square tests and multivariate logistic regression were employed to assess associations between clinical variables and complication risk. Time-to-resolution of complications was analyzed using Kaplan-Meier (KM) survival curves. Relative risks (RR) were computed for each surgical incision type.

Results

ZMC fractures predominantly affected males (n=69, 90%), most frequently in the 20-29-year age group (n=22, 28.6%). Road traffic accidents, assaults, and falls were the primary causes. Maxillary fractures were most common (n=25, 32.4%), and the buccal sulcus incision was preferred (n=36, 46.7%). Infraorbital paresthesia was the most frequent complication (n=5, 27%). Significant predictors of complications included age ≥50 years, infraorbital fractures, infraorbital incisions, and alcohol-related trauma (p < 0.05). KM analysis revealed delayed resolution of paresthesia and diplopia; hemianopia persisted at 12 weeks. Overall, open reduction and internal fixation (ORIF) demonstrated a favorable safety profile.

Conclusions

ZMC fractures primarily affect young adult males and are most often trauma-induced. While ORIF remains the standard for managing displaced fractures, increased complication risks in older adults and alcohol-related injuries highlight the need for individualized surgical planning and targeted postoperative care.

## Introduction

The zygomatic bone, or zygoma, serves as a critical component of the midfacial skeleton, conferring facial width, structural support to the orbital framework, and acting as an essential buttress for mastication and facial expression [1]. As the principal midfacial buttress, the zygoma not only contributes to craniofacial aesthetics but also maintains functional integrity through its articulation with adjacent bones and its role as a key attachment site for masticatory muscles. Owing to its prominent anatomical position, the zygoma is highly susceptible to trauma, and zygomaticomaxillary complex (ZMC) fractures represent the second most common maxillofacial injuries, accounting for approximately 13% of all craniofacial fractures [2,3]. The primary etiological factors include road traffic accidents, interpersonal violence, falls, and sports-related incidents.

Clinically, ZMC fractures may manifest as minor contour irregularities or progress to significant functional impairments, including periorbital ecchymosis, infraorbital nerve dysfunction, enophthalmos, trismus, and ocular motility restriction [4]. Untreated or inadequately managed ZMC fractures can result in long-term sequelae such as facial asymmetry, chronic pain, and persistent masticatory dysfunction. Timely surgical intervention, particularly within two weeks of injury, is crucial for optimizing both functional and aesthetic outcomes, with delayed management being associated with increased risk of unfavorable results [4].

Contemporary management strategies for ZMC fractures have evolved to incorporate both open and closed reduction techniques, with fixation methods tailored according to the severity and complexity of the fracture. Fixation strategies, including one-point, two-point, and three-point approaches, are selected based on the extent of displacement and the involvement of the orbital floor [5]. Common surgical approaches comprise transconjunctival, subciliary, intraoral, and lateral eyebrow incisions, each offering distinct advantages in terms of access, exposure, and postoperative morbidity.

Despite advances in surgical techniques, the long-term stability and relapse rates associated with closed reduction methods, such as the Gillies approach, remain a subject of ongoing debate. While minimally invasive approaches offer advantages of reduced morbidity and operative time, concerns persist regarding their capacity to achieve and maintain anatomical reduction, particularly in cases of complex or comminuted fractures. Currently, there is limited evidence on the comparative effectiveness of closed versus open reduction and internal fixation (ORIF) in optimizing clinical outcomes and minimizing complications in ZMC fracture management.

Accordingly, the present retrospective study was designed to systematically evaluate the demographic distribution, injury mechanisms, fracture patterns, surgical approaches, management strategies, and associated postoperative complications among patients with ZMC fractures treated at our institution over a three-year period. The central aim of this study is to provide evidence-based insights to guide the optimization of treatment protocols for maxillofacial trauma. The null hypothesis (H₀) tested is that there is no significant association between the choice of management strategy and the incidence of postoperative complications or clinical outcomes in ZMC fractures.

## Materials and methods

Study design

The study was conducted at the Department of Oral and Maxillofacial Surgery, RVS Dental College and Hospital, Coimbatore, Tamil Nadu. This study was designed as a single-center, retrospective, descriptive analysis of patients treated for ZMC fractures. The study was conducted at a tertiary care dental center with institutional review board approval (RIDC/IEC/I/08/2024) in accordance with the ethical principles outlined in the Declaration of Helsinki (2013 revision). Written informed consent was obtained from all patients for the use of their anonymized medical records in research.

Patient selection

The study included all patients presenting with craniomaxillofacial trauma between January 2021 and December 2024 whose final diagnosis confirmed a ZMC fracture. Medical records, operative notes, and imaging studies were reviewed to identify eligible cases. The inclusion and exclusion criteria for including the patients in the study are presented in Table 1.

**Table 1:** Inclusion and exclusion criteria

Category	Criteria
Inclusion	1. Patients aged ≥ 18 years with a confirmed diagnosis of zygomaticomaxillary complex (ZMC) fracture based on clinical and radiographic findings.
	2. Patients treated for craniomaxillofacial trauma at the study institution between 2021 and 2024 with a minimum of one-year postoperative follow-up.
Exclusion	1. Incomplete medical or imaging records.
	2. Associated polytrauma involving other facial regions.
	3. Pathological fractures or malignancy.
	4. Follow-up period less than one year.

Clinical and demographic data collection

Patient demographics (age, sex), mechanism of injury (e.g., motor vehicle accidents, assault, falls), fracture classification (according to the Zingg et al. system [6]), surgical approach, incision type, management strategy (conservative vs. surgical), fixation method (closed reduction or ORIF), and postoperative complications were systematically recorded. Data were extracted from medical charts, operative notes, and radiologic images by two independent reviewers to ensure accuracy and consistency.

Fracture classification and management

ZMC fractures were classified using the Zingg et al. system, which stratifies fractures by anatomical displacement and severity [6]. Management strategies were categorized as conservative (non-surgical) or surgical intervention. Surgical interventions were further subdivided into closed reduction and ORIF, with incision type and fixation method documented. The choice of approach was determined by fracture severity, displacement, and surgeon preference.

Postoperative follow-up and outcome assessment

All patients underwent standardized postoperative follow-up for a minimum of one year. Clinical outcomes, including functional recovery, restoration of facial symmetry, and resolution of symptoms, were assessed at regular intervals. Postoperative complications, such as infraorbital paresthesia, diplopia, edema, and vision disturbances, were recorded at each follow-up visit.

**Figure 1:**
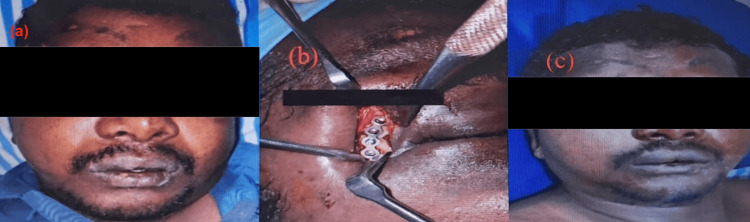
Clinical presentation and surgical management a: clinical finding showing right periorbital edema; b: ORIF of right frontozygomatic suture; c: postoperative picture ORIF: open reduction and internal fixation Written informed consent to include this image in an open-access article was obtained from the patient.

Figure 1 illustrates the clinical presentation and surgical management of a patient with a right frontozygomatic fracture, including periorbital edema at presentation (Figure 1a), intraoperative ORIF (Figure 1b), and the postoperative outcome (Figure 1c).

**Figure 2:**
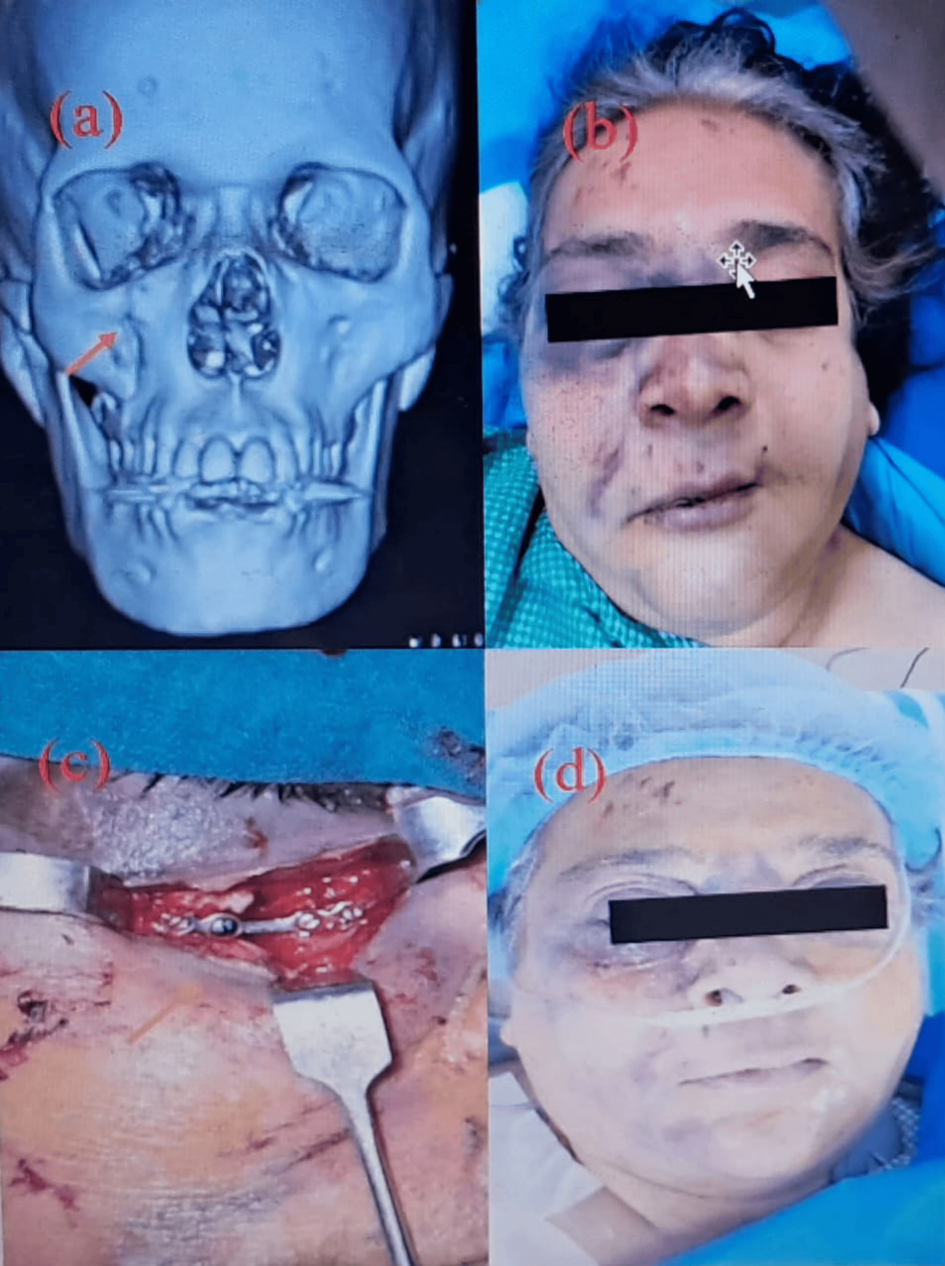
Clinical presentation and surgical management a: 3D reconstructed CT facial shows a right infraorbital fracture (red arrow); b: clinical finding showing right Raccoon's eye, subconjunctival ecchymosis; c: ORIF of right infraorbital fracture (arrow); d: postoperative picture ORIF: open reduction and internal fixation Written informed consent to include this image in an open-access article was obtained from the patient.

As shown in Figure 2, the patient exhibited a right infraorbital fracture confirmed on CT (Figure 2a), accompanied by raccoon’s eye and subconjunctival ecchymosis (Figure 2b); ORIF was performed (Figure 2c), with favorable healing observed in the postoperative period (Figure 2d).

**Figure 3:**
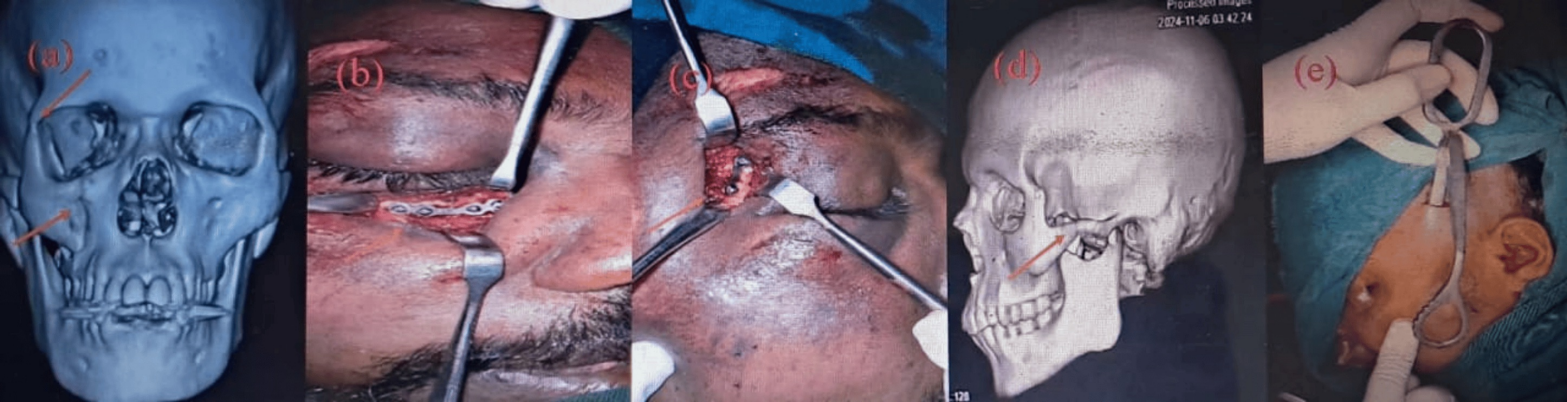
Management of a complex fracture a: 3D reconstructed CT facial showing right infraorbital and frontozygomatic suture fracture (red arrow); b, c: ORIF under GA; d: 3D reconstructed CT facial showing displaced zygomatic arch fracture (red arrow); e: closed reduction of Zygoma using Gilles' temporal approach ORIF: open reduction and internal fixation; GA: general anesthesia Written informed consent to include this image in an open-access article was obtained from the patient.

Figure 3 demonstrates the management of a complex fracture involving both infraorbital and frontozygomatic regions seen on CT imaging (Figure 3a), managed surgically under general anesthesia (Figures 3b, 3c); additionally, a displaced zygomatic arch fracture (Figure 3d) was addressed via closed reduction using the Gilles temporal approach (Figure 3e).

**Figure 4:**
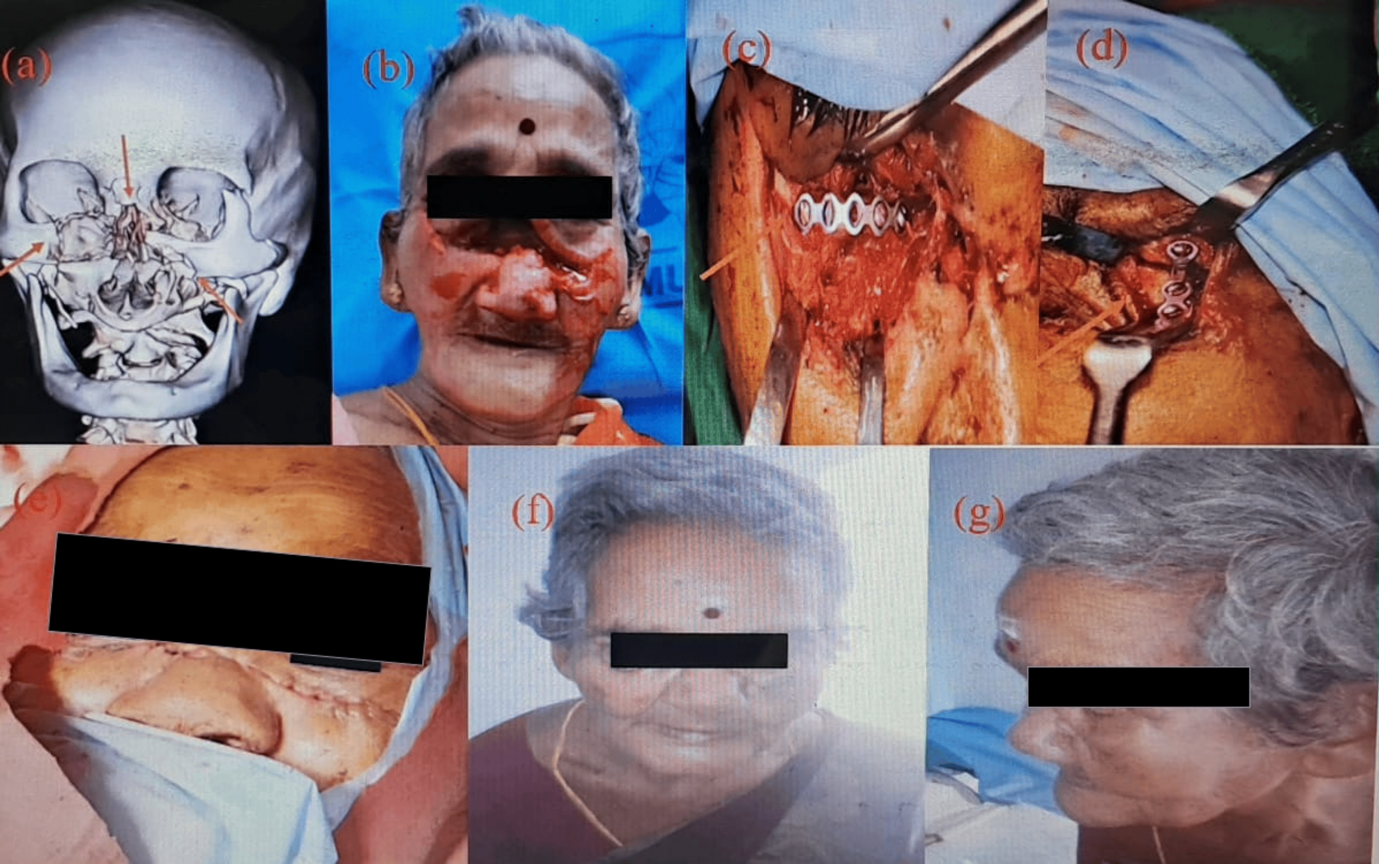
Multiple midfacial fractures a: 3D reconstructed CT facial showing multiple midface fractures (red arrows); b: clinical picture showing deep horizontal laceration; c, d: ORIF under GA (arrow); e: postoperative picture; f, g: one-month follow-up ORIF: open reduction and internal fixation; GA: general anesthesia Written informed consent to include this image in an open-access article was obtained from the patient.

A case of multiple midfacial fractures is depicted in Figure 4, highlighting CT-confirmed injury (Figure 4a), associated deep horizontal laceration (Figure 4b), intraoperative ORIF (Figures 4c, 4d), and favorable outcomes at postoperative review (Figure 4e) and one-month follow-up (Figures 4f, 4g).

Sample size estimation

A priori sample size calculation was performed using the “Exact - Proportion: Difference from constant (binomial test, one sample case)” method. With a one-tailed test, effect size g = 0.15, alpha = 0.05, power (1-β) = 0.85, and a constant proportion of 0.3, the minimum required sample size was 77 patients, providing an actual post-hoc power of 0.86 and an alpha error of 0.0492.

Statistical analysis

All data analyses were performed by an independent investigator using IBM SPSS Statistics for Windows, Version 27 (Released 2019; IBM Corp., Armonk, New York, United States). Demographic variables, including patient age, sex, incidence, and classification of ZMC fractures, surgical approaches, incision type, and postoperative complications, were summarized as frequencies and percentages. The distribution of categorical variables across subgroups was evaluated using the chi-square test. In particular, associations between mode of injury and age group were examined using chi-square analysis. Multivariate logistic regression was employed to identify predictors of postoperative complications following ZMC fracture repair, and results were reported as adjusted odds ratios (aOR) with 95% confidence intervals (CI). Kaplan-Meier (KM) survival analysis was used to estimate the time-to-resolution of postoperative complications, with survival probabilities calculated at four, eight, and 12 weeks. Relative risks (RR) for complication occurrence based on surgical incision type were computed using the buccal sulcus approach as the reference. The Katz method with continuity correction was applied to derive 95% CIs for RR. Fisher’s exact test and Holm-adjusted p-values were used for multiple comparisons. For all comparisons, a p-value < 0.05 was considered indicative of statistical significance.

## Results

Study population

Over the three-year study period (2021-2024), a total of 280 patients with craniomaxillofacial injuries were screened. After the application of inclusion and exclusion criteria, 77 patients with confirmed ZMC fractures were included in the final analysis (Table 2).

**Table 2:** Demographic data ZMC: zygomaticomaxillary complex

Study variables	Frequency	Percentage distribution (%)
Age group (years)		
<19	6	7.8
20-29	22	28.6
30-39	18	23.4
40-49	16	20.8
50-59	12	15.6
60-69	2	2.6
>70	1	1.2
Gender		
Male	69	90
Female	8	10
Incidence of ZMC fractures		
Nasal	11	14.3
Infraorbital	18	23.4
Frontozygomatic	15	19.5
Maxilla	25	32.4
Isolated zygoma	8	10.4
Types of incision used		
Lateral eyebrow	15	19.5
Infraorbital	18	23.4
Buccal sulcus	36	46.7
Keen’s approach	2	2.6
Gilles temporal incision	6	7.8

The majority of patients were male (n=69; 90%), with females accounting for only 10% (n=8) of the cohort. The 20-29-year-old age group comprised the largest proportion of cases (n=22, 28.6%), followed by patients aged 30-39 years (n=18, 23.4%) and 40-49 years (n=16, 20.8%). The proportion of older participants progressively declined, with only a single case (n=1, 1.2%) identified in individuals above 70 years of age.

Fracture classification and surgical approaches

Maxillary fractures represented the most frequent fracture pattern (n=25, 32.4%), followed by infraorbital (n=18, 23.4%) and frontozygomatic (n=15, 19.5%) fractures. Nasal involvement was documented in 14.3% (n=11) of cases, while isolated zygomatic fractures were the least common (n=8, 10.4%). Regarding surgical management, the buccal sulcus incision was the most commonly employed approach (n=36, 46.7%). Less frequently utilized techniques included the Gilles temporal incision (n=6, 7.8%) and Keen’s approach (n=2, 2.6%).

Complications and postoperative outcomes

Postoperative complications were observed in nine out of 77 patients, corresponding to a relatively low incidence. Infraorbital paresthesia was the most common complication, affecting five patients (n=5, 27%), primarily attributed to nerve manipulation or entrapment during reduction procedures. Lymph edema occurred in two patients (n=2, 11%), typically manifesting on the third postoperative day and resolving within eight days. Diplopia was also noted in two patients (n=2, 11%), with all cases managed successfully using corticosteroid therapy. Importantly, no instances of hemianopia or vision loss were recorded, indicating a low rate of severe visual complications (Table 3).

**Table 3:** Postoperative complications

Complications	No. of cases (9)	Percentage (%)
Infraorbital paresthesia	5	27
Lymph edema	2	11
Hemianopia	0	0
Diplopia	2	11

Associations between age and injury mechanism

Chi-square analysis of the distribution of injury mechanisms by age group revealed a statistically significant association (chi-square test statistic (χ²)=42.5, degree of freedom (df)=24, effect size (Cramer's V=0.33, p=0.020) (Table 4).

**Table 4:** Chi-square test performed for mode of injury in specific age groups *p<0.05 is statistically significant; **p<0.01 is statistically highly significant

Age groups	<19		20-29		30-39		40-49		50-59		60-69		>70		P-value
	n	%	n	%	n	%	n	%	n	%	n	%	n	%	
Sports	1	16.67	2	9.09	0	0.00	0	0	0	0.00	0	0	0	0	0.020*
Motor vehicle collision (not by alcohol)	2	33.33	12	54.55	6	33.33	6	37.5	7	58.33	1	50	0	0	
Under alcohol influence	2	33.33	6	27.27	5	27.78	8	50	5	41.67	1	50	1	100	
Fall by others	0	0.00	1	4.55	6	33.33	0	0	0	0.00	0	0	0	0	
Assaults	1	16.67	1	4.55	1	5.56	2	12.5	0	0.00	0	0	0	0	

Among individuals under 19 years, sports injuries (n=1, 16.7%) and motor vehicle collisions (n=2, 33.3%) were common, with a similar proportion (n=2, 33.3%) related to alcohol influence. In the 20-29 year group, motor vehicle accidents predominated (n=12, 54.6%), followed by alcohol-related trauma (n=6, 27.3%). Falls were most frequently observed in the 30-39 year group (n=6, 33.3%), while injuries under the influence of alcohol were the leading cause in the 40-49 year age group (n=8, 50%). Among patients aged 50-59 years, motor vehicle collisions (n=7, 58.3%) remained the principal etiology, with a substantial proportion of alcohol-related cases (n=5, 41.7%). Notably, all ZMC fractures in participants (n=1, 100%) above 70 years were attributed to alcohol influence.

Multivariate predictors of postoperative complications

Multivariate logistic regression analysis revealed that increasing age was associated with a progressively higher likelihood of postoperative complications following ZMC fracture management (Table 5). Compared to patients under 19 years of age, those aged 50-59 years exhibited a significantly increased risk (adjusted odds ratio (aOR)=7.10; 95% CI: 1.46-34.45; p=0.015). Similarly, individuals aged 60-69 years (aOR=6.92; 95% CI: 1.03-46.58; p=0.047) and those older than 70 years (aOR=9.83; 95% CI: 1.16-83.16; p=0.036) also showed significantly elevated risks. Age groups between 20-49 years did not reach statistical significance (p > 0.05). Gender did not significantly influence complication rates, with females showing a nonsignificant increase in risk compared to males (aOR=1.22; 95% CI: 0.26-5.65; p=0.801). Among fracture types, infraorbital fractures were significantly associated with complications (aOR=5.56; 95% CI: 1.56-19.89; p=0.008), whereas frontozygomatic, nasal, and isolated zygoma fractures did not show significant associations (p > 0.05 for all).

**Table 5:** Multivariate logistic regression predicting postoperative complications in ZMC fracture patients NS: not significant; ZMC: zygomaticomaxillary complex; MVC: motor vehicle collision

Predictor	Adjusted odds ratio (aOR)	95% CI	p-value	Interpretation
Age group (Ref: <19 years)				
20–29	1.42	0.21–9.50	0.728	NS
30–39	1.59	0.24–10.53	0.630	NS
40–49	2.22	0.36–13.80	0.392	NS
50–59	7.10	1.46–34.45	0.015	Significant
60–69	6.92	1.03–46.58	0.047	Significant
>70	9.83	1.16–83.16	0.036	Significant
Gender (Ref: Male)				
Female	1.22	0.26–5.65	0.801	NS
Fracture type (Ref: Maxilla)				
Infraorbital	5.56	1.56–19.89	0.008	Significant
Frontozygomatic	1.64	0.36–7.38	0.519	NS
Nasal	0.95	0.17–5.19	0.952	NS
Isolated zygoma	0.61	0.07–5.21	0.658	NS
Incision (Ref: Buccal sulcus)				
Infraorbital	4.75	1.36–16.54	0.015	Significant
Lateral eyebrow	1.61	0.39–6.66	0.510	NS
Keen’s approach	2.07	0.18–23.49	0.563	NS
Gilles temporal incision	0.74	0.09–6.04	0.783	NS
Mode of injury (Ref: Sports)				
MVC (not by alcohol)	1.98	0.31–12.68	0.464	NS
Alcohol	6.30	1.21–32.96	0.029	Significant
Fall by others	1.02	0.08–12.56	0.985	NS
Assaults	2.11	0.18–24.70	0.557	NS

The type of surgical incision also influenced outcomes. Infraorbital incisions significantly increased the odds of postoperative complications compared to the buccal sulcus approach (aOR=4.75; 95% CI: 1.36-16.54; p=0.015). Other incisions, including lateral eyebrow, Keen’s approach, and Gilles' temporal, did not show significant associations.

 Regarding mode of injury, alcohol-related injuries were significantly associated with complications (aOR=6.30; 95% CI: 1.21-32.96; p=0.029), while other causes, such as motor vehicle collisions not involving alcohol, falls, or assaults, were not significant predictors (p > 0.05) (Table 5).

Time-to-resolution of postoperative complications

KM survival analysis was performed to evaluate the resolution of specific postoperative complications over a 12-week period (Table 5). Among the assessed complications, infraorbital paresthesia persisted in the majority of patients, with only 6.5% achieving resolution by 12 weeks. Diplopia and lymph edema each showed a 2.6% resolution rate. Hemianopia showed no resolution within the 12-week period. The survival estimates indicated a high probability of complications remaining unresolved at 12 weeks (e.g., S(12w)=0.935 for paresthesia, 0.974 for edema, 1.000 for hemianopia) (Table 6).

**Table 6:** Time-to-resolution analysis of postoperative complications following zygomaticomaxillary complex (ZMC) fracture management

Complication	N	Events (resolved)	Censored	S(4w)	S(8w)	S(12w)	Resolved by 12w (%)
Infraorbital paresthesia	77	5	72	1.000	0.974	0.935	6.5
Lymph edema	77	2	75	1.000	0.974	0.974	2.6
Hemianopia	77	0	77	1.000	1.000	1.000	0.0
Diplopia	77	2	75	1.000	0.987	0.974	2.6

**Figure 5:**
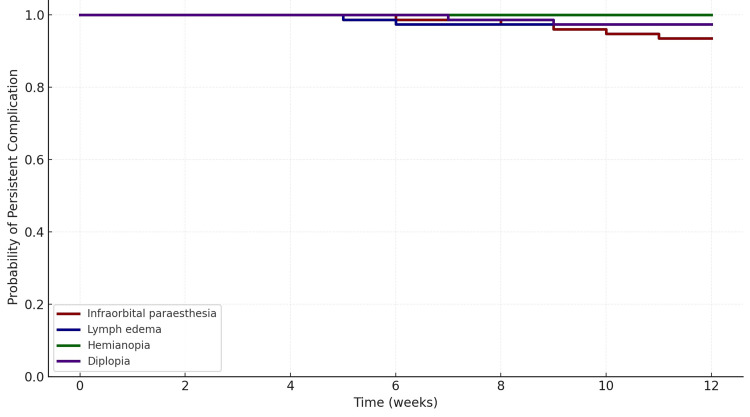
Kaplan-Meier graph for resolution of complications

S(t) is the KM estimate of the probability that the complication is still present at time t (four, eight, 12 weeks). \[\text{Resolved by 12w (\%)} = 100 \times \left[ 1 - S(12w) \right]\] The KM plot displays the time-to-resolution of four postoperative complications following ZMC fracture surgery over 12 weeks (Figure 5). The y-axis represents the probability of persistent complication (i.e., the complication has not yet resolved), while the x-axis denotes the follow-up time in weeks.

Among the complications, hemianopia (green line) showed no resolution in any patient throughout the 12-week period, remaining at a constant survival probability of 1.0, indicating complete persistence. Infraorbital paresthesia (dark red line) showed the greatest rate of persistence, with a gradual decline in survival probability over time. By week 12, it dropped to approximately 0.935, indicating that around 6.5% of cases had resolved, while the vast majority remained unresolved. Diplopia (purple line) demonstrated slightly better resolution, with a survival probability of ~0.974 at week 12, corresponding to a 2.6% resolution rate. A similar pattern was observed for lymph edema (dark blue line), which also had a 2.6% resolution by the end of follow-up.

Association between surgical incision and complication risk

When comparing surgical approaches, the risk of postoperative complications varied by incision type (Table 7). The buccal sulcus approach, used in 36 patients, had the lowest observed complication rate (5.6%) and served as the reference. The infraorbital approach showed a higher complication rate (22.2%) with an RR of 4.00 (95% CI: 0.81-19.82), but this did not reach statistical significance (p=0.087; Holm-adjusted p=0.349). Similarly, the lateral eyebrow and Gilles temporal incisions had higher risk estimates (RR=2.40 and 3.00, respectively), though these findings were not statistically significant (p > 0.05 for both). The Keen’s approach showed no complications in the two patients treated with this method, yielding an RR estimate that was not statistically meaningful due to the small sample size. The overall chi-square test for association between incision type and complications was not significant (χ²=3.695, effect size (Cramér’s V)=0.219, df=4; p=0.475), indicating that while trends suggest increased risk with infraorbital access, the evidence does not support a strong relationship when controlling for multiple comparisons (Table 7).

**Table 7:** Association between surgical incision type and postoperative complications in ZMC fractures Overall association: χ² (df=4)=3.695; pperm=0.475; Cramér’s V=0.219. Relative risk (RR) values were calculated using the buccal sulcus approach as the reference. 95% confidence intervals for RR were computed using the Katz method with a continuity correction applied for cells with zero events. ZMC: zygomaticomaxillary complex

Incision type	Complications (Yes) (n)	Complications (No) (n)	Total (n)	Risk (%)	Relative risk (RR) vs. buccal sulcus	95% CI	p(Fisher)	p (Holm-adjusted)
Buccal sulcus	2	34	36	5.6	Reference	–	–	–
Infraorbital	4	14	18	22.2	4.00	0.81–19.82	0.087	0.349
Lateral eyebrow	2	13	15	13.3	2.40	0.37–15.50	0.571	1.000
Gilles temporal	1	5	6	16.7	3.00	0.32–28.17	0.378	1.000
Keen’s approach	0	2	2	0.0	2.47	0.15–40.53	1.000	1.000

## Discussion

This retrospective observational study investigated demographic and etiological patterns, fracture types, surgical approaches, treatment strategies, and postoperative complications in patients undergoing management for ZMC fractures. The analysis revealed notable trends in age-related risk, fracture patterns, and surgical approaches, with particular emphasis on predictors and time-to-resolution of postoperative complications.

The present data confirm that males were predominantly affected, reflecting established trends observed in trauma epidemiology and attributed to greater exposure to high-risk activities, occupational hazards, and substance use. These findings are consistent with previous reports, including the national epidemiological data from Patil et al. [7], which highlight road traffic accidents as the leading cause of ZMC fractures in India. The high incidence is driven by rapid urbanization, deficient enforcement of road safety laws, and poor compliance with protective measures such as helmets and seat belts [8-17].

The pattern of fractures observed in our cohort aligns with the existing literature, where tripod fractures remain the most common subtype, consistent with the reports of Zingg et al. (57%) and Ashwin et al. (54.35%) [6,10]. Nearly one-third of patients sustained concomitant craniofacial fractures, particularly mandibular fractures, which occurred in 44% of these cases. This finding correlates with biomechanical studies indicating that mandibular involvement is often seen in high-energy trauma due to force transmission through the facial skeleton.

Management decisions in ZMC fractures are guided by the degree of displacement, functional deficits, and cosmetic concerns. In our study, ORIF was employed in 89% of cases, which is in line with previous retrospective analyses that report surgical management rates of approximately 84% [9]. The Gilles temporal and Keen’s intraoral approaches were reserved for zygomatic arch fractures, enabling adequate access while minimizing tissue morbidity. For non-displaced or minimally displaced fractures, closed reduction was performed in 16% of cases, confirming the role of conservative strategies under appropriate indications, as advocated by Lee et al. [11].

The infraorbital, buccal sulcus, and lateral eyebrow incisions were the most frequently used exposure techniques in our sample. Notably, multivariate analysis identified infraorbital incisions as significantly associated with postoperative complications compared to buccal sulcus incisions. However, other surgical approaches did not exhibit statistically significant associations. These findings resonate with the results of Crosara et al. [17], who noted more visible scarring with infraorbital access but no significant increase in periorbital complications. Additionally, Momeni Roochi et al. [16] found no significant differences in complications such as entropion or ectropion between transconjunctival and subciliary incisions.

Age emerged as a critical predictor of complications, with patients over 50 years experiencing significantly higher odds of postoperative issues. These results reinforce the importance of considering systemic factors such as vascularity, bone quality, and nerve susceptibility in elderly individuals undergoing facial trauma surgery.

Complication-specific KM analysis demonstrated that infraorbital paresthesia was the most frequently encountered postoperative complication, with a resolution rate of only 6.5% at 12 weeks. Despite most cases resolving within three to six months during long-term follow-up, early persistence of sensory deficits underscores the need for careful intraoperative handling of the infraorbital nerve. Diplopia and lymph edema also showed delayed resolution, while hemianopia exhibited no resolution during the 12-week observation period, suggesting the possibility of permanent optic nerve damage in select cases. Corticosteroid therapy, including tapering regimens of methylprednisolone, was effective in managing transient diplopia and visual loss in the few affected patients.

Importantly, alcohol-related injuries were significantly associated with increased postoperative complications, reflecting both the severity of trauma and the potential confounding role of delayed hospital presentation and impaired healing capacity in these patients. These findings emphasize the importance of tailored perioperative management and counselling in patients with alcohol-related facial trauma.

Although the RR of postoperative complications appeared higher in patients treated with infraorbital and eyebrow incisions, the overall association between incision type and complications did not reach statistical significance, potentially due to limited sample sizes in some groups. A larger cohort may help delineate the true effect of incision choice on complication risk.

Several limitations of the present study warrant consideration. First, the retrospective nature and single-center design may limit generalizability, and prospective studies are necessary to confirm these findings. Second, although a one-year follow-up was maintained, only early resolution data (up to 12 weeks) were analyzed using KM estimates. Longitudinal survival analyses beyond three months would better capture the full course of sensory and visual recovery. Third, while statistical models accounted for age, gender, fracture type, incision, and etiology, unmeasured confounders such as surgeon experience and intraoperative complications could have influenced outcomes.

These findings underscore the importance of patient-specific treatment planning, especially in older adults and individuals presenting with infraorbital fractures or alcohol-related trauma. Infraorbital access, though effective for exposure, should be undertaken with caution, given the associated risk of nerve injury. Conservative reduction techniques remain viable in select fracture types, especially arch injuries, when performed using established approaches like the Gilles technique. Future studies should focus on long-term sensory recovery, functional rehabilitation outcomes, and patient-reported quality of life, which will help refine the selection of optimal surgical strategies for ZMC fracture management.

## Conclusions

Within the limitations of this study, ZMC fractures were most common in young adult males, primarily from road traffic accidents. Conservative management sufficed for non-displaced fractures, while ORIF, commonly via the buccal sulcus approach, provided optimal outcomes for displaced cases. Although transient complications such as infraorbital paresthesia and diplopia were noted, age above 50 years and alcohol-related injuries significantly increased complication risks. These findings underscore the importance of individualized management and preventive measures, including stricter road safety enforcement and public awareness, to reduce facial trauma incidence.
